# Fear, xenophobia and collectivism as predictors of well-being during Coronavirus disease 2019: An empirical study from India

**DOI:** 10.1177/0020764020936323

**Published:** 2020-07-04

**Authors:** Kanika K Ahuja, Debanjan Banerjee, Kritika Chaudhary, Chehak Gidwani

**Affiliations:** 1 Department of Psychology, Lady Shriram College for Women, University of Delhi, Delhi, India; 2 Department of Psychiatry, National Institute of Mental Health and Neurosciences (NIMHANS), Bengaluru, India

**Keywords:** Well-being, xenophobia, fear of coronavirus, collectivism, COVID-19

## Abstract

**Background:**

The Coronavirus disease 2019 (COVID-19) has emerged as a global health threat. Biological disasters like this can generate immense prejudice, xenophobia, stigma and othering, all of which have adverse consequences on health and well-being. In a country as diverse and populous in India, such crisis can trigger communalism and mutual blame. Keeping this in context, this study explored the relationship between well-being and xenophobic attitudes towards Muslims, collectivism and fear of COVID-19 in India.

**Methods:**

The study was carried out on 600 non-Islamic Indians (231 males, 366 females and 3 others; mean age: 38.76 years), using convenience sampling. An online survey containing Fear of Coronavirus scale, Warwick–Edinburgh Mental Well-Being Scale and Collectivism Scale was used. Xenophobia was assessed using two scales: generalized prejudice towards Muslims and specific xenophobic tendencies towards Muslims during COVID-19. The data were analysed using correlational methods and multiple regression.

**Results:**

The findings showed that positively significant relationship exists between well-being and age as well as with collectivism, while an inversely significant relationship between well-being and fear of COVID-19 was found. The results of the multiple regression analysis shows that fear of COVID-19, age, collectivism and generalized xenophobia, in the order of their importance, together contributed to nearly 20% of variance in well-being.

**Conclusion:**

The findings are reflective of the importance of collectivism in enhancing well-being in these times of uncertainty. Xenophobia, one of the common offshoots of pandemics, can also harm the overall well-being. Implications are discussed in the light of India’s diverse socio-religious background and global context.

## Introduction

The Coronavirus disease 2019 (COVID-19), a ‘Public health emergency of International Concern’, as declared by the World Health Organization (WHO), has affected nearly every country irreversibly. As of 2 June 2020, 213 countries/territories had more than 6 million confirmed cases and around 4,00,000 deaths (WHO, 2020). The only possible way so far to contain the spread of this contagious disease is the imposition of lockdown to maintain social distancing. Thus, over 100 countries of the world (including India) experienced a total or partial lockdown from March 2020. Due to the highly contagious nature of this disease and the resulting lockdown, it is natural that the fear associated with the disease is also rising, affecting the well-being of people. Research in the Indian context showed that the anxiety levels in the sample were high with more than 80% sample preoccupied with thoughts of coronavirus (Roy et al., 2020). Sleep difficulties (12.5%), paranoia about getting infected with COVID-19 (37.8%) and distress-related social media (36.4%) were reported. A review of the psychological impact of quarantine reported negative psychological effects, including posttraumatic stress symptoms, confusion and anger. Some of the notable stressors included fears of infection, frustration, boredom, inadequate supplies, inadequate information, financial loss and stigma (Brooks et al., 2020).

Prior evidence also suggests that the outbreak of other infectious diseases (like Severe Acute Respiratory Syndrome (SARS)) led to significantly lower levels of subjective well-being (Lau et al., 2008). Important linkages between anxiety and depression and viral diseases such as influenza A (H1N1) have been reported (Coughlin, 2012). Social distancing through the first wave of the 2009 influenza A (H1N1) pandemic in Hong Kong was also found to be associated with higher anxiety, poorer health, greater perceived risk of infection and greater worry of becoming ill (Cowling et al., 2010).

Fear is the cradle for interpersonal hatred and social stigma. WHO has identified this pattern and has given guidelines for managing fear and discrimination in COVID-19. Certain sections of the society especially the vulnerable populations have been targeted in the outbreak. India is no exception to this pattern. Considering its varied socio-cultural and religious diversity, it stands at a unique risk of prejudice and xenophobia during pandemics. For months now, we have been consuming news and opinions about COVID-19. Much of the news in India has centred around the Tablighi Jamaat, an Islamic missionary movement, which held a congregation at its global headquarters in Delhi even after the announcement of the lockdown in the country. This turned out to be the major potential source of the spread of coronavirus across the country as many of the Jamaat members who returned to their hometowns at the end of March carried the infection to their cities and villages, and thus became the vectors of the virus. A statement by the Union Ministry of Health and Family Welfare in India declared that there has been a surge of 30% in the cases of coronavirus because of the Tablighi Jamaat. The description of the spread of COVID-19 as the ‘Tablighi Spread’ has been as divisive as attempts to call COVID-19 as the ‘Wuhan virus’ or Kung Flu. A video advising people not to buy vegetables from Muslim vendors and posters on fruit shops in Jamshedpur to demarcate the faith of shopkeepers have stoked communal cauldron (Singh, 2020; TNN Agencies, 2020), although the Government authorities took suitable action later. The United Nations (UN) Commission on International Religious Freedom downgraded India into its list of ‘Country of Particular Concern’ because of alleged policies and treatment towards the Muslim population, a charge that is vehemently denied by the Government (The Times of India, 2020). The Census of India (2011) reports Hinduism (80%) and Islam (14%) as two major religions in the country. Due to various factors like fear of terrorism, the political dispute over Kashmir, international unrest and internal social dynamics, Islamophobia has emerged as a prominent xenophobic construct in India (Sanke et al., 2018). Further bigoted opinions, fake news by media and viral videos have resulted in stigmatization of COVID-19 in India. It becomes all the more evident during such public health crisis and adds to the psychosocial burden as well as quality of life.

Islamophobia over recent years has reached record highs in some Western countries as well (Allen & Nielsen, 2002; Amnesty International, 2012; Bleich, 2009) over increased terror attacks, immigration and demographic shifts. Research has pointed out a relationship between xenophobia and well-being. Subjective ill-being (opposite of subjective well-being) has been associated with Islamophobia in Germany (Sirgy et al., 2019). The National Well-Being Index in South Africa was reported to have a statistically significant relationship with attitudes towards immigrants (Gordon, 2015). Such prejudice often induces mass agitation, violence, mutual blame and hysteria, all of which can be risk factors for psychological stress and trauma. Studying prejudicial attitudes can promote other social benefits and is therefore of critical importance.

Xenophobic tendencies become all the more evident during the spread of infectious diseases. One such explanation is provided by the disease-avoidance model. This model links the adaptive utility of avoiding harmful pathogens and parasites with contemporary prejudices against individuals who are perceived to be potential carriers of pathogens or parasites (Kurzban & Leary, 2001; Park et al., 2003). In line with the disease-avoidance model, it was shown that chronic and contextually aroused feelings of vulnerability to disease motivated negative reactions to foreign immigrants (Faulkner et al., 2004). Temporary exposure to pathogen cues (e.g. reading news of swine flu) increases prejudice towards real-world immigrants (Huang et al., 2011). Another formulation, the pathogen prevalence hypothesis, helps to understand the relation between disease threat, collectivism and xenophobic tendencies. According to this hypothesis, people living in regions with a high prevalence of pathogens show increased collectivistic behaviours. This leads to greater conformity and higher xenophobia (Murray et al., 2013). One investigation on xenophobia and the threat of Ebola reported that the more vulnerable people felt to Ebola, the more they exhibited prejudice towards West Africans and immigrants, but this relationship was moderated by individualism and collectivism (Kim et al., 2016). Also, during the earlier outbreak of SARS, prejudice and othering have been mentioned as offshoots of public behavior (Blendon et al., 2004). Similarly, evidence of a positive association between countrywide measures of pathogen prevalence, collectivism and xenophobia has been provided (Fincher et al., 2008).

To date, little is known about the interrelations between xenophobia and prejudice (in this case towards Muslims, seen as potential carriers of COVID-19) and collectivism, in the light of fear of COVID-19, and how this will affect the well-being of people. The present study thus aimed to explore the relationship between xenophobic attitudes towards Muslims, collectivism, fear of COVID-19 and well-being in India.

## Methods

### Participants

The sample comprised of 600 participants (231 males, 366 females and 3 others), between the age of 18 and 83 years, from 26 states/union territories (UTs) representing nearly all parts of India. Inclusion criteria were as follows: (1) an Indian national who identified himself or herself as non-Muslim currently residing in India, (2) aged 18 years or older and (3) able to understand English. All participants belonged to middle- or upper-class income strata. Those who identified themselves as Muslims were excluded from the study.

Socio-demographic characteristics of the sample were collected through a specially designed demographic data sheet. Their mean (±*SD*) age was 38.76 ± 15.87 years. Majority of the participants (86.8%) were Hindus, 4.8% Sikhs, 2.8% Christians and the remaining 5.6% belonged to other religious faiths; 293 participants (48.8%) were employed, 139 participants (23.2%) were unemployed (largely homemakers or retired) and 168 (28%) were students; 342 (57%) were married, 210 (35%) were single and 6 (1%) were in a live-in relationship; 539 participants (89.8%) were living with someone and 61 participants (10.2%) were living alone; 203 (33.8%) reported education as their nature of work followed by 72 (12%) homemakers, 60 (10%) in business, 52 (88.7%) professionals and the remaining were in health services, real estate, finance, retired and so on.

### Procedure

The present study was conducted at Lady Shri Ram College for Women. All procedures performed in studies involving human participants were in accordance with the formal ethical standards of Lady Shri Ram College for Women, as recommended by the University of Delhi, India (University of Delhi, 2020). Participants were recruited through convenience sampling. They were contacted via email, various WhatsApp groups (for instance, Resident Welfare Associations of several localities) and several posts on Facebook. Care was taken to accommodate responses from all parts of India. A Google form was created for online administration. Participants were told that the purpose of the study was to understand personal values of people, and how people perceive others from different social backgrounds in the midst of COVID-19 pandemic. After ensuring confidentiality of responses, all the tools were administered. Socio-demographic information about their gender, religion, age, occupation, nature of work, city, relationship status and living arrangement was also sought. Participation was voluntary, and no monetary compensation was given. All participants provided electronic informed consent before taking part in this study.

### Instruments

#### Fear of Coronavirus scale

This seven-item scale is designed to assess fear of COVID-19 among the general population (Ahorsu et al., 2020). Example items include the following: ‘I am most afraid of coronavirus’, ‘It makes me uncomfortable to think about coronavirus-19’ and ‘My hands become clammy when I think about coronavirus-19’. The participants indicate their level of agreement with the statements using a 5-point Likert-type scale. Answers included ‘strongly disagree’, ‘disagree’, ‘neither agree nor disagree’, ‘agree’ and ‘strongly agree’. The minimum score possible for each question is 1, and the maximum is 5. A total score is calculated by adding up each item score (ranging from 7 to 35). Authors reported a mean of 27.39 for 717 Iranian participants. The item-total correlation ranged from .47 to .56. Reliability values such as internal consistency (α = .82) and test–retest reliability (intraclass correlation coefficient (ICC) = .72) were acceptable. Concurrent validity was supported by the Hospital Anxiety and Depression Scale (with depression, *r* = .425 and anxiety, *r* = .511) and the Perceived Vulnerability to Disease Scale (with perceived infectability, *r* = .483 and germ aversion, *r* = .459). We created a composite, with higher scores indicating greater fear of COVID-19 (α = .848).

#### Warwick–Edinburgh Mental Well-Being Scale

Warwick–Edinburgh Mental Well-Being Scale (WEMWBS) is a 14-item scale of positively worded statements covering feelings and functioning aspects of mental well-being (Tennant et al., 2007). The 14 items have five response categories from ‘none of the time’ to ‘all of the time’. Example items include the following: ‘I’ve been feeling useful’ and ‘I’ve been feeling relaxed’. Each question uses a 5-point response scale, ranging from 1 (*none of the time*) to 5 (*all of the time*). Test–retest reliability at 1 week was high (.83). It correlates highly with similar tests, such as WHO-Five Well-Being Index (*r* = .77**) and Short Depression Happiness Scale (*r* = .76**). Social desirability bias was lower or similar to that of other comparable scales (Tennant et al., 2007). The authors report a very high level of internal consistency (*r* = .94), which was found in the present study as well (α = .926). Total scores range from 14 to 70. The Scottish population mean reported by the authors is 50.7 with a 95% confidence interval.

#### Collectivism scale

An eight-item measure was used to assess an individual’s level of collectivism (Kim et al., 2016). Participants indicated how much they agreed or disagreed with the statements, using a scale from 1 (*strongly disagree*) to 7 (*strongly agree*). These statements were drawn from scales assessing collectivism (α = .81). Example item for collectivism was ‘Learning about the traditions, customs, values, and beliefs of my family is important to me’. The total scores range from 8 to 56. The authors report a high level of internal consistency (*r* = .833). We created a composite, with higher scores indicating greater collectivism (α = .833).

#### Xenophobia scale

Xenophobia was assessed with two indicators. The first indicator, generalized xenophobia, assessed generalized prejudice towards Muslims. This six-item scale requires participants to rate their feelings (three negative: hostility, disliking and fear; three positive: acceptance, sympathy and warmth) towards the Muslims on scales ranging from 0 (*I do not feel this emotion at all*) to 7 (*I feel this emotion strongly*) (Fincher et al., 2008). Positive emotion items were reverse-scored, such that higher scores indicate greater negativity. The scores ranged from 0 to 42. The authors report a very high level of internal consistency (α = .72 for prejudice towards West Africans and α = .81 for prejudice towards immigrants). In the present study, we found a similar Cronbach’s alpha (α = .724).

The second indicator, specific xenophobia, was developed by the present authors to assess the xenophobic tendencies towards the Muslims specifically during the times of COVID-19 pandemic in the Indian context; 10 items were given in a 5-point response format ranging from 1 (*strongly disagree*) to 5 (*strongly agree*). Example item includes the following: ‘The outbreak of COVID-19 in India is primarily due to Muslims’. Two items (‘Hindus/other religious groups are just as responsible for transmission of COVID-19 as Muslims’ and ‘The media should not be allowed to paint Muslims in poor light’) were reverse-scored. Total score ranges from 10 to 50. A higher score is indicative of high xenophobic tendencies towards Muslims in the context of COVID-19 in India (α = .88).

### Statistical analysis

Data were analysed using the SPSS 22.0. Associations of age, fear of coronavirus, collectivism and xenophobia with well-being were assessed using Pearson’s correlation coefficient. Then, multiple regression was done to study variables which were able to predict well-being during times of the pandemic. A *p* value of <.05 was considered statistically significant.

## Results

The data analysis was carried out using the scores from the completed surveys. Descriptive statistics, Pearson’s correlation and multiple regression were utilized for analysis.

Table 1 shows the descriptive statistics of the study variables. Participants indicated a low fear of COVID-19 (17.05 ± 5.96) when compared to an Iranian population (Ahorsu et al., 2020): above average collectivism levels (36.76 ± 8.96), below average generalized xenophobia (15.04 ± 8.54) and above average specific xenophobia (26.47 ± 8.91). Furthermore, participants reported an average level of well-being (51.33 ± 10.10). The value obtained for skewness indicates a symmetric distribution for all the variables, while that for kurtosis implies a distribution that is neither too peaked nor too flat.

**Table 1.** Descriptive statistics of the study variables.

Factors	Minimum	Maximum	Range	*M*	*SD*	Skewness *SE* = .100	Kurtosis *SE* = .199
Age	16.00	83.00	67.00	38.768	15.867	0.338	−0.842
Fear of COVID-19	7.00	35.00	28.00	17.047	5.964	0.375	−0.236
Well-being	14.00	72.00	58.00	51.335	10.099	−0.529	0.634
Collectivism	8.00	56.00	48.00	36.765	8.963	−0.287	−0.176
Generalized xenophobia	0.00	42.00	42.00	15.040	8.537	0.313	−0.238
Specific xenophobia	10.00	49.00	39.00	26.475	8.910	0.210	−0.628

*SE*: standard error; COVID-19: Coronavirus disease 2019.

The results of Pearson’s correlation coefficients in Table 2 reveal pertinent relationships between age and well-being (*r* = .332, *p* < .05), between fear of COVID-19 and well-being (*r* = –.235, *p* < .05), between generalized xenophobia and well-being (*r* = –.010, *p* > .05), between specific xenophobia and well-being (*r* = .132, *p* < .05), between age and collectivism (*r* = .344, *p* < .05) and between collectivism and fear of COVID-19 (*r* = .106, *p* < .05).

**Table 2.** Correlation indices for the relationship among study variables.

Factors	1	2	3	4	5	6
Age	1	.006	.332**	.344**	.174**	.274**
Fear of COVID-19		1	−.235**	.122**	.106**	.132**
Well-being			1	.295**	−.010	.132**
Collectivism				1	.249**	.478**
Generalized xenophobia					1	.573**
Specific xenophobia						1

COVID-19: Coronavirus disease 2019.

** Correlation is significant at the .01 level (two-tailed). Correlation is significant at the .05 level (two-tailed).

Table 3 reveals a significant effect of fear of COVID-19, age, collectivism, generalized xenophobia and specific xenophobia on well-being of non-Muslim population (*R*^2^ = .225, *F* = 34.625, *p* < .01). This indicates that age, fear of COVID-19, collectivism and generalized xenophobia account for 22.5% of the variance in well-being in the context of the pandemic.

**Table 3.** Model summary and coefficients of collectivism, fear of COVID-19, age, generalized xenophobia and specific xenophobia on well-being.

Factors	Unstandardized coefficients		Standardized coefficients	*T*	Significance
	*B*	*SE*	Beta		
(Constant)	43.020	1.843		23.248	.000
Collectivism	0.277	0.048	.246	5.767	.000
Fear of COVID-19	−0.440	0.062	−.260	−7.096	.000
Age	0.163	0.025	.257	6.608	.000
Generalized xenophobia	−0.135	0.052	−.114	−2.580	.000
Specific xenophobia	0.050	0.055	.044	0.893	.372

COVID-19: Coronavirus disease 2019; *SE*: standard error.

Model summary *R* = .475, *R*^2^ = .225, *R*^2^ (adjusted) = .219, *F* = 34.625, *p* < .01.

Predictors: (constant), collectivism, fear of COVID-19, age, generalized xenophobia and specific xenophobia.

Dependent variable: well-being.

Among all the variables, the most potent contributor to well-being is fear of COVID-19 (β = –.26, *t* = –7.096, *p* < .05), although negative followed by age (β = .257, *t* = 6.61, *p* < .05); collectivism (β = .246, *t* = 5.77, *p* < .05) was the next potent predictor followed by generalized xenophobia (β = –.114, *t* = –2.580, *p* < .05). Specific xenophobia was not found to contribute to well-being (β = .044, *t* = 0.893, *p* > .05).

## Discussion

The results of the present study showed that fear of COVID-19, age, collectivism and generalized xenophobia are closely linked with well-being. As expected, a significant negative relationship between fear of COVID-19 and well-being was found. Fear of COVID-19 was also the most potent contributor in determining well-being. Insufficient information regarding the cause of the disease, the exact reason for onset of the pandemic, its rapid transmission, lack of cure and vaccine, and high levels of uncertainty has led to an increased fear among the people across the globe. Immediate changes like self-quarantine, social distancing, spread of rumours via social media, burden of misinformation and restriction in travelling added to the fear and stress, thereby leading to worry and restlessness which adversely affects well-being (Banerjee, 2020).

Results suggest a positive and significant relationship between well-being and collectivism. Collectivism was also found to predict well-being. Collectivistic tendencies – feeling of belongingness, greater strength of social connections and importance given to needs of one’s family – act as buffer against the high levels of uncertainty and stress accompanying the spread of infectious diseases. This forms the basis for resilience and acts as a protector against the viral infection (Kim et al., 2016; Murray et al., 2013), at least in collectivistic societies like India where ‘joint families’ and ‘social connectedness’ are thought to be protective factors for interpersonal coherence. Previous research has also suggested that social relationships are an effective way of reducing the counterproductive defensiveness as well as overreactions in anxiety provoking situations involving the spread of infectious diseases (Sherman & Cohen, 2006). Family connections indeed are an important social resource that fosters well-being. They affirm against the threat of the disease and associated fear of isolation, and provide a sense of purpose, security and meaning, more so in tough times (Ryff, 2014). Furthermore, a significant positive relationship between well-being and age (*r* = .33, *p* = .000) was also found. It has been reported that older people are more collectivistic and group-oriented than younger ones (Chen, 2015), which may have contributed to their well-being and resilience. Moreover, due to an extended lockdown, the elderly are getting to spend more time with their families, leading to a rise in perceived social support. It is also possible that since germ aversion and perceived vulnerability to disease increase as both men and women get older (Díaz et al., 2020), they are staying more at home, a practice that is contributing to their well-being.

Interestingly, the results of the multiple regression suggest that possessing negative attitudes towards Muslims leads to decreased well-being. This is consistent with previous research (Gordon, 2015; Sirgy et al., 2019). The mean rating on xenophobia measures shows that specific xenophobia (blaming some Muslims miscreants for the spread of COVID-19) was above average. Blaming ‘the other’ has been often cited as a way to make mysterious and devastating diseases comprehensible and possibly controllable (Nelkin & Gilman, 1988). On the contrary, encouragingly, generalized xenophobia, emotions of hostility, prejudice and interpersonal disgust, contempt and anger towards the Muslims were below average. This is in line with Cohn (2012), who surveyed the history of pandemics in the West, and contested long-held assumptions that epidemics sparked blame of the ‘Other’, and that it was worse when diseases were mysterious as to their causes and cures. He concluded that blame and hate were rarely connected with pandemics (Cohn, 2012).

It may also be contended that those high on xenophobia may see a greater threat of spread of COVID-19 by Muslims, which further increases their fear of COVID-19, leading to reduced well-being. A similar finding was reported by researchers who conducted their research at the height of H1N1 swine-flu epidemic (Huang et al., 2011). They demonstrated that when threatened with disease, vaccinated participants exhibited less prejudice towards immigrants than unvaccinated participants did. They also found that having some participants simply washing their hands for hygienic needs significantly influenced participants’ perceptions of out-group members.

The results of the present study therefore suggest that to improve well-being, fear of COVID-19 should be addressed. This will also help to reduce xenophobic tendencies. At the same time, collectivistic beliefs and social support mechanisms should be strengthened. Recommendations that reduce people’s fears of getting infected with the COVID-19, such as practicing good sanitation habits, regular hand washing, precautions when buying groceries and wearing of masks, should be reinforced. At the same time, proactive steps towards assuring public should be taken. Perhaps the low mortality rates of COVID-19, despite its highly infectious nature, could be highlighted. Due to the bulk of ‘information overload’ during this pandemic, the numbers of recoveries are often neglected under the burden of fatalities. This will result in an enhanced sense of protection from the disease, which will not only foster well-being but also reduce bias towards people who are not legitimate carriers of disease. Such bias has been present in the Indian society ever since the ‘divide and rule’ policy of the British Raj and subsequently the partition (Kulkarni, 2018). Crisis and disasters can easily incite this ‘social evil’.

Media need to cover more positive stories such as members of the Tablighi Jamaat, an Islamic organization, volunteered to donate blood for plasma therapy into COVID-19 patients (The Times of India, 2020). The negative effects of prejudice and discrimination have been shown to disturb psychological and physical well-being among targets of prejudice (Lewis et al., 2011). The present study shows that holding xenophobic attitudes may be harmful not just for the target of prejudice but also for those holding such attitudes. Understanding how to improve prejudicial attitudes is therefore of critical importance in enhancing well-being.

One of the limitations in our study was that since data were collected from the middle/upper middle strata of the Indian society, no claims can be made about generalizability of results to other income groups. It is also possible that the respondents’ responses could have been influenced by social desirability factors. Since fear of COVID-19, collectivism, age and generalized xenophobia towards Muslims accounted for only 22.5% of variance in the well-being, other factors may be relevant too. Also, the Fear of COVID-19 Scale (Ahorsu et al., 2020) has not yet been validated in India. However, considering the lack of availability of any other relevant scales and the items of the scale being generic and socio-culturally non-specific, we chose to use this for the study. Future research could study other such factors as the frustration and anxiety induced due to boredom of not being able to carry with the daily routine while in quarantine, having inadequate basic supplies during quarantine, as contributing to well-being (Wilken et al., 2017). Finally, the importance of individual’s cultural orientation (individualism/collectivism) in predicting well-being differs among societies/countries. While in the present Indian sample, collectivism has been found to be related to well-being, whether the same applies in other nations as well is not certain, and future research is required. Also, the authors do not intend to suggest that stigma against the religious group studied here is specific to the adverse consequences on well-being. The authors imply that xenophobia against any community or ethnic/religious minorities can affect overall health, especially during times of such crises. We chose this particular Islamic and non-Islamic groups here considering the prevalence of the two major religious sectors in India.

## Conclusion

Well-being is negatively affected with fear of COVID-19 and generalized xenophobia. Age and collectivism are found to be significantly positively related with well-being. Holding xenophobic attitudes towards any religious community (Muslims in the study) is not just bigotry, it also reduces psychological well-being. Bulk of misinformation, rumour-mongering, negative perceptions and role of media are important to influence the prejudice and stigma during pandemics. The case fatality is surely a concern. However, such mutual hatred and communalism can further increase public agitation and competition for health care, overburdening the limited health resources in the country. Stakeholders at all levels, individual, community and administrative, need to be responsible for preventing this. At times when the world is facing an unprecedented threat, preventing any form of marginalization can improve positivism and resilience. The more COVID-19 is stigmatized, the more divisive, inflammatory and counterproductive it will be. ‘Collective’ connectedness can help humanity live and emerge through this pandemic, perhaps stronger and more hopeful than before.
